# Temporally Coherent Synchrotron Light Sources

**DOI:** 10.1107/S1600577526004212

**Published:** 2026-04-23

**Authors:** Sergey Antipov, Ilya Agapov, Ralf Röhlsberger, Hans-Christian Wille

**Affiliations:** a Deutsches Elektronen-Synchrotron (DESY), Hamburg, Germany; b Helmholtz Institute Jena Jena Germany; ESRF – The European Synchrotron, France

**Keywords:** temporally coherent synchrotron light sources, X-ray FEL oscillator (XFELO), self-seeding, storage rings, microbunching, undulator radiation

## Abstract

The workshop *Temporally Coherent Synchrotron Light Sources* was held at DESY, Hamburg, Germany on 26–27 January 2026 to examine scientific drivers, accelerator concepts and a community pathway toward longitudinally coherent X-ray sources.

## Abstract 2

Report on the *Temporally Coherent Synchrotron Light Sources* workshop, held at DESY, Hamburg, Germany, 26–27 January 2026.

The workshop *Temporally Coherent Synchrotron Light Sources* was held at DESY (Hamburg, Germany) on 26–27 January 2026 as a satellite to the annual DESY/European XFEL Photon Science Users’ Meeting (see https://indico.desy.de/event/51344/ for program and materials). More than 50 participants from the photon science, free-electron laser and storage-ring-based light source communities gathered together to address the following question: how can we exploit synergies between light sources and free-electron lasers for the next generation of photon science facilities?

Today’s and planned fourth-generation diffraction-limited storage rings, such as ESRF–EBS, SOLEIL II, HEPS and PETRA IV, feature a high degree of transverse radiation coherence for user experiments. With this push towards diffraction limit we are approaching the fundamental limits of transverse coherence, and the next frontier is gaining systematic control over longitudinal (temporal) coherence. At the same time, the rapid progress of X-ray free-electron lasers (FELs) presents both complementarity and competition with storage rings, motivating renewed efforts to extend temporal coherence in high-repetition-rate synchrotron sources. Exploiting synergies between storage rings, noted for stability and high average flux, and FELs, characteristic of ultrashort pulses and high peak brightness, one can extend the accessible time–frequency phase space for photon science (H. Tanaka, SPring-8, Japan).

The key question is to identify the scientific potential offered by temporally coherent X-rays. From a user’s perspective, ‘temporal coherence’ is not an abstract source property but a concrete requirement: reproducible spectral brightness and phase stability over many pulses, enabling rapid averaging and correlation-based methods. One concrete science-case example is incoherent diffractive imaging (IDI), where stable, high-repetition beams make it practical to extract three-dimensional structural information from intensity correlations of incoherently scattered (often fluorescent) X-rays without relying on first-order phase coherence. Another example is nuclear resonant scattering and related nuclear quantum optics. For instance, the narrow-bandwidth photon delivery to resonant excitation of the ^45^Sc nuclear clock isomer with a natural linewidth Γ_0_ ≃ 1.4 × 10^−15^ (Yu. Shvyd’ko, ANL, USA) sharpens the performance targets and illustrates the spectral selectivity that is expected from temporally coherent sources. Further science drivers include nonlinear X-ray optics, where tailored multi-pulse timing structures and stable spectra could move the field beyond proof-of-principle demonstrations, as well as molecular and soft-matter dynamics, where MHz-class probing promises access to regimes currently limited by repetition rate and stability.

Realization of such a source is not a trivial task and three families of routes toward enhanced longitudinal coherence were explored. First, cavity-based concepts, in particular X-ray FEL oscillators (XFELOs), present a path to near-transform-limited hard X-ray pulses at high repetition rate, provided that stringent requirements on cavity thermomechanics and timing synchronization can be met. Recent progress toward an XFELO demonstrator at the European XFEL (P. Rauer, DESY, Germany) serves as a practical anchor for debates on realistic cavity performance, outcoupling, and operational constraints such as heat load on Bragg crystals. Second, seeding techniques present a spectrum of increasingly mature tools: self-seeding is routinely deployed at hard X-ray FELs to reduce bandwidth and improve spectral density, while externally seeded operation is advancing toward fully coherent soft X-ray pulses. From the storage-ring perspective, echo-enabled harmonic generation (EEHG) in the DELTA storage ring (S. Khan, TU Dortmund, Germany) is an existence proof for exporting laser coherence to shorter wavelengths. Third, microbunching-based schemes in circular machines offer a route to combining coherence enhancement with inherently high repetition rate; steady-state microbunching (SSMB) concepts and experiments highlighted both the potential of quasi-continuous coherent emission and the demanding need for turn-by-turn phase control, tight tolerances, and careful handling of ring lattice nonlinearities.

An important common requirement for achieving temporal coherence is maintaining high-intensity beams under control. From an accelerator physicist’s point of view, coherence-enhanced operation is ultimately an operating-point problem: narrow bandwidth and stable phase must be delivered together with sufficient photon flux while preserving lifetime, stability and machine protection margins. Modern low-emittance storage rings such as ESRF–EBS gained substantial operational experience relevant to coherent operation. It emphasizes the tension between short, dense bunches favourable for coherent emission and high harmonics and collective effects that increase energy spread, drive instabilities, and degrade beam lifetime (S. White, ESRF, France). In this context, microbunching-related dynamics plays a dual role: often detrimental to X-ray spectral quality (yet exploitable as a source of bright coherent THz) and as sensitive diagnostics of longitudinal phase space.

In conclusion, the workshop demonstrated that, as storage rings approach their emittance limits, future gains in scientific capability increasingly depend not only on brightness but also on spectral purity, phase stability and pulse structure. Progress toward temporally (longitudinally) coherent synchrotron light sources will likely proceed along multiple complementary paths: incremental coherence improvements at existing FELs and rings; targeted demonstrators (such as cavity FEL and microbunching testbeds); and longer-term designs that merge high repetition rate with narrow bandwidth and stability, including possible ring-based oscillator concepts. ▸

## Figures and Tables

**Figure 1:**
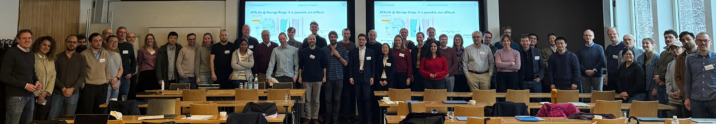
Group photograph of 50 participants representing the storage ring, FEL and photon science communities at the *Temporally Coherent Synchrotron Light Sources* workshop (DESY, Hamburg, Germany; 26–27 January 2026).

